# Barriers to HIV testing among male clients of female sex workers in Indonesia

**DOI:** 10.1186/s12939-018-0782-4

**Published:** 2018-05-30

**Authors:** Nelsensius Klau Fauk, Anastasia Suci Sukmawati, Pius Almindu Leki Berek, Ernawati, Elisabeth Kristanti, Sri Sunaringsih Ika Wardojo, Isaias Budi Cahaya, Lillian Mwanri

**Affiliations:** 1 Institute of Resource Governance and Social Change, Jl. R. W. Monginsidi II, No. 2, Kupang, Nusa Tenggara Timur 85221 Indonesia; 2 Stikes Jenderal Achmad Yani Yogyakarta, Jl. Ringroad Barat Ambarketawang, Gamping, Sleman, Yogyakarta, 55294 Indonesia; 3 Jurusan Keperawatan, Universitas Timor, Jl. Wehor Kabuna Haliwen, Atambua, NTT, 85711 Indonesia; 4 Sekolah Tinggi Ilmu Kesehatan Sint Carolus, Jl. Salemba Raya 41, Jakarta, 10440 Indonesia; 5 Timor University, Jl. Km 09, Kelurahan Sasi, Kefmenanu, NTT, 85613 Indonesia; 6 Universitas Muhammadiyah Malang, Jl. Raya Tlogomas, Malang, 65144 Indonesia; 7 Samuel J. Moeda Indonesian Navy Hospital, Jl. Yos Sudarso No.5 Osmok Kupang, Nusa Tenggara Timur, 85232 Indonesia; 8 College of Medicine and Public Health, Flinders University, GPO Box 2100, Adelaide, South Australia 5001 Australia

**Keywords:** Barriers, HIV testing, Male clients of FSWs, Indonesia

## Abstract

**Background:**

Frequent engagement of men in sexual encounters with female sex workers (FSWs) without using condoms places them at a high risk for HIV infection. HIV testing has been noted to be among important strategies to prevent HIV transmission and acquisition. However, it is known that not all men willingly undertake an HIV test as a way to prevent HIV transmission and/or acquisition. This study aimed to identify barriers to accessing HIV testing services among men who are clients of FSWs (clients) in Belu and Malaka districts, Indonesia.

**Methods:**

A qualitative inquiry employing face to face open ended interviews was conducted from January to April 2017. The participants (*n* = 42) were clients of FSWs recruited using purposive and snowball sampling techniques. Data were analysed using a qualitative data analysis framework.

**Results:**

Findings indicated three main barriers of accessing HIV testing services by clients. These included: (1) personal barriers (lack of knowledge of HIV/AIDS and HIV testing availability, and unwillingness to undergo HIV testing due to low self-perceived risk of HIV and fear of the test result); (2) health care service provision barriers (lack of trust in health professionals and limited availability of medication including antiretroviral (ARV)); and (3) social barriers (stigma and discrimination, and the lack of social supports).

**Conclusions:**

These findings indicated multilevelled barriers to accessing HIV testing services among participants, who are known to be among key population groups in HIV care. Actions to improve HIV/AIDS-related health services accessibility are required. The dissemination of the knowledge and information on HIV/AIDS and improved available of HIV/AIDS-related services are necessary actions to improve the personal levelled barriers. System wide barriers will need improved practices and health policies to provide patients friendly and accessible services. The societal levelled barriers will need a more broad societal approach including raising awareness in the community and enhanced discussions about HIV/AIDS issues in order to normalise HIV in the society.

## Background

Male clients of female sex workers (FSWs) (here on to be known as clients) as well as the FSWs themselves have been indicated as at high risk groups for HIV infection [1]. Studies with this group in different settings have reported that HIV infection is highly prevalent among them [2–6]. Frequent engagement in sexual encounters with multiple FSWs without condoms has been incriminated as one of the main contributors to the transmission of HIV within this population group [7–10]. Sexual intercourse has been reported as one of the main routes of HIV spread in Indonesia, and men including these clients compared to women, are more susceptible to the infection with 59.2 and 40.8% respectively in last 5 years [11].

Promoting access to HIV testing as well as voluntary counselling among individuals, including clients of FSWs, at risk for HIV infection has been considered as a key strategy for HIV prevention and associated with reduced risk behaviour [12–14]. The World Health Organization has also recommended members of key populations, including clients of FSWs, who are at high risk for HIV infection to undergo HIV testing at least once a year [15]. However, studies and reports have shown that access of the clients of FSWs to HIV testing in many settings is still low [16–20]. Barriers to accessing HIV testing services among these clients have been reported elsewhere. These include: (i) clients’ fear of HIV diagnosis which has been associated with stigma and discrimination, (ii) fear of losing a position in the community due to societal norms of masculinity which position men as physically strong (and HIV being associated with weakness), (iii) fear of losing the ability to be self-reliant (having HIV being associated with having AIDS which could lead to loss of the ability to work), (iv) clients failing to make time for testing, and (v) the denial about being vulnerable to HIV, including low self-perception of HIV risk [17, 19, 21, 22].

To our knowledge, the study participants, clients of FSWs, have not been the target of HIV prevention programs or interventions in the study settings as well as in Indonesia [11]. Likewise, accessibility to HIV testing services and barriers to undergoing HIV testing among clients in this nation have not been well documented. Based on the educational backgrounds and professional experiences in the field of HIV/AIDS including in the study settings, the study authors had prior knowledge of HIV testing barriers described above, and including: the lack of knowledge and information on HIV/AIDS, poor availability and or accessibility of HIV/AIDS-related health services, stigma and discrimination, and overall poor transportation to and from the healthcare facilities. This qualitative inquiry aimed to identify and document barriers to accessing HIV testing services among a sample of clients in Malaka and Belu districts, Indonesia.

## Methods

### Theorectical framework

The current study was guided by access of health service (i.e.HIV testing service in this case) framework [23]. This framework identifies five dimensions of accessibility of health service including:approachability, acceptability, availability, affordability, and appropriateness) [23]. For example, approachability of services may relate to services being well known by participants and whether participants feel welcomed by the service providers. Acceptability may refer to the cultural and social aspects that influence the individual’s acceptance of care, and which is judged by the person’s perceptions of the appropriateness of the care. Availability may mean that the service exists and can be reached in a timely manner. This may be argued in relation to physical availability of the HIV testing service in this study settings and that male clients of FSWs have the ability to know the existence of these services and to access them regularly for effective diagnosis of their HIV status. Affordability refers to the economic capacity of clients of FSWs to pay for transportation and HIV testing service. It also reflects the availability time to travel and access the service. Appropriateness would relate to whether the care meets the participants’ needs, including whether the clients trust the professionals or care providers. Additionally, the access framework further identifies the following five abilities of the populations that correspond with the dimensions of accessibility: ability to perceive (perception of the need of some service due to literacy level or cultural perceptions); ability to seek (could be influenced by personal values or knowledge of existence of the service); ability to reach (issues including, transport availability, cost and the way in which the services operates- e.g. issues whether professionally or whether clients would feel stigmatised and develop fear of their status been known to the wide community) [23]. Ability to pay (for instance, the affordability of services is dependent on the cost of the services as well as the clients’ ability to pay for the services and ability to engage (e.g. when the services were not culturally appropriate to meet issues of clients of FSWS in Indonesia).

### Study setting and design

The selection of the study settings was based on several factors including professional experiences of the researchers especially NKF and PALB who reside in the study districts. All researchers are health professionals including medical doctors, nurses and health practitioners, and are experienced researchers in public health. The principal author (NKF) was an experienced manager of HIV/AIDS programs targeting different population groups in the study settings and the senior author (LM) is a public health physician who chairs several committees related to migration, public health and chronic disease including HIV/AIDS. Additionally, previous studies in the same districts [24, 25] provided information regarding the existence of high numbers of local and nonlocal FSWs.

A qualitative inquiry was conducted from January to April 2017 to identify barriers to accessing HIV testing among male clients of FSWs from several rural areas in Belu and Malaka districts, Indonesia. The use of a qualitative study design was useful in providing the researchers with opportunities to have direct interactions with the study participants and observe the situations and setting where they lived, worked and interacted [26]. Because sex work is illegal, FSWs and their clients have not been the target groups for HIV services [24, 25] in these districts or in Indonesia. The two study districts can be considered as representative for the province as they have socio-economic, cultural and religious norms and values similar to the other districts in East Nusa Tenggara province. The study participants (*n* = 42) were male clients of FSWs recruited purposively and additionally using a snowball sampling technique. The first stage was purposive where a staff member of a non-governmental organisation (NGO) that provides HIV/AIDS services in the districts and known to researchers was contacted to enlist his help. Four initial participants known to be clients of FSWs were provided with the study information by this staff member. These four initial participants were then asked to snowball and circulate the study information including researchers’ contact details to other people who were potential study participants. The inclusion criteria for participation were: (a) individuals who aged 18 years old or above, and (b) a male client of FSWs. Following the dissemination of information as describe above, 18 potential participants who met the inclusion criteria contacted the researchers during the following 3 weeks. All of them consented to be interviewed voluntarily. After each interview, participants were asked to distribute the same information sheet to people they knew to be clients of FSWs. A further 20 participants consented to participate in the study.

Face to face open ended interviews were conducted to collect the data from the participants using an interview schedule. The development of the interview was informed by the access of health service theoretical framework described above. The guide explored broader issues including: factors that made the HIV testing services accessible to participants: e.g. knowledge about HIV/AIDS and HIV testing and HIV treatment issues; availability of HIV testing and HIV medications; and other socio-cultural issues such as cost of the service and medications, attitudes and behaviours of health professionals. Although thematic questions derived from this framework were employed to guide the interviews, we maintained the essence of in-depth interview technique by having open ended questions for participants to provide their broader experiences (than would be the framework) of HIV testing services accessibility in these settings. A total of 42 clients of FSWs were interviewed to reach data saturation [27] where no further new information seemed to emerge. Interviews with each participant were scheduled at their convenient time and place and were conducted in Bahasa. All consenting participants responded to all questions and none of participants withdrew from the interviews.

### Data analysis

The recorded data were transcribed into coding sheets and translated into English by the research team.Although feedback from participants was not sought after transcriptions, to maintain the quality and validity of the data, data were crossed checked and comparisons between the researchers were conducted during the transcription and translation process.

The qualitative data analysis framework by Ritchie and Spencer [28, 29] was employed to analyse the data. This framework offereda systematic approach to manage the data and provided coherence and structure of analysis [30–32]. The data analysis followed five steps including:

(i) *Familiarisation* with the data through reading transcripts line by line repeatedly, breaking data into several chunks and commenting or labelling the data;
(ii) *Identifying* key issues, concepts and themes in a coding frame and developing a coding scheme;
(iii) *Indexing* the entire data by creating open coding where similar and redundant codes were identified and the list made smaller and manageable. This was followed by creating closed coding where codes referring to the same theme were grouped together. This stage led to development of 11 codes which were further synthesised to shortlist three overarching themes for the current paper;
(iv) *Charting* the data which involved arranging appropriate thematic references in a summary chart to enable comparisons across the interviews and within each interview;
(v) *Mapping and interpretation* which enabled examination of ideas that made up the themes, and for relationship and association between them to be seen. The framework analysis approach enhances rigour, transparency and validity to the analytic process [33]. Analysis was deductively (categories derived from the researchers’ prior knowledge on the topic and the theoretical framework) and inductively (categories emerging purely from the data) [34].

## Results

### Participants’ profile (Table 1)

A total of 42 participants were involved in this study. Participants’ ages ranged between 19 and 35 years, with the majority of them being single (93%). None of the study participants reported to have had HIV testing at the time of interviews. Participants reported multiple barriers to accessing HIV testing services and these are summarised in three themes (personal, health service and social determinants), and further elaborated below.

**Table 1:** Socio-demographic characteristics of the participants

Characteristics	No. of Respondents *N* = 42 (%)
Age	
19 - 25	16 (38)
26 - 30	18 (43)
31 - 35	8(19)
Job	
Motorcycle taxi drivers (*Ojek*)	15 (36)
Construction workers	14 (33)
Port workers	10(24)
Unemployed	3 (7)
Education	
High school graduates	35(83)
Elementary school graduates	7(17)
Condom use with FSWs	
Never	18 (43)
Sometimes	16 (38)
Always	8 (19)
Number of sexual encounters with FCSWs in the past 6 months	
1 – 5 times	5 (12)
6 – 10 times	11 (26)
≥ 11 times	26 (62)
Number of FSWs they had sex with in the past 6 months	
2 - 5	13 (31)
6 - 10	22 (52)
≥ 11	7 (17)

### Personal barriers

#### Lack of knowledge of HIV testing and HIV/AIDS service and lack of knowledge of the existence of HIV infection

Although HIV testing service was made available in hospitals and community health centres in the districts for all to utilise, findings of the current study indicate that none of the study participants had undergone HIV testing. Lack of knowledge of the availability of this health service seemed to be among the barriers to accessing this service for participants across age and work groups. A total of 24 participants stated that they were not aware of these services. Assertions from a few participants’ testimonials are depicted below:

“I have been living here for the whole of my life and visited our community health centre too many times but I am not aware of free HIV testing provided by the [local] government ....Nobody told me, the health professionals in the community health centre did not say anything about it” (P11: 19 years old).

“.... The testing might be available at the community health centre but I haven’t heard of such service .... I have often accessed health services at our community health centre but not that one [HIV testing], I do not know anything about it” (P20: 22 years old).

“I never take HIV test because I do not know. Staff from the community health centre often visit communities here but they do not talk about the [HIV] test, they do not tell us to do it” (P32: 23 years old).

“I do not know anything about it [HIV testing service], maybe because I hardly go to hospitals. Based on my experience, it seems like information about the test does not exist, I have a lot of friends but nobody talks about it, or they might not know about it either ....” (P39: 25 years old).

The lack of general knowledge about HIV/AIDS services and the lack of knowledge about the existence of HIV infection were barriers for taking HIV prevention measures among participants. For example, a failure to HIV testing meant that clients of FSWs did not know about their HIV status. In the current study, most participants acknowledged that they were unaware of the existence of HIV/AIDS, which seemed to be one of the factors precluding them from accessing available HIV testing services. The testimonials below allude to these assertions:

“I am not a highly educated person, just an elementary school graduate. I do not read newspapers or articles or book. I know nothing about HIV/AIDS and never think of HIV testing either” (P3: 20 years old).

“I would have looked for the [HIV] test if I knew about this disease [HIV/AIDS] and that I could be infected as well. How could I know if nurses and midwives do not provide us information about the disease? I am busy with my work every day, from morning till evening ....” (P7: 28 years old).

“I think it is possible for me to do the [HIV] test but how can I test for HIV if I am not aware that HIV/AIDS exists? I do not know about the disease and the test. Every day I try to earn money, never think of diseases” (P15: 31 years old)

“I never heard of it [HIV/AIDS]. There is no information about that disease in the place like this [the harbour], everybody is busy with loading and unloading the materials. I have not tested for HIV because I do not know about the test and HIV ....” (P28: 21 years old).

### Unwillingness to undergo HIV testing and perceived seriousness of HIV infection

Unwillingness to undergo HIV testing was found to be an important hindering factor among the clients who were aware of the availability of free HIV testing as well as counselling services in the districts. Low self-perceptions of the risk of acquiring HIV infection seemed to be one of the reasons that made them unwilling to take the test to discover their HIV status:

“I have heard of the [HIV] test once I attended the HIV information session but I feel okay so far, nothing to worry about. I do not think I need to take HIV test because I am physically fine, I do not have any physical complaints” (P4: 22 years old).

“I do not have sex that often with them [FSWs], only once or twice in a month ....I think I am not at risk of getting HIV infection, so I do not want to test for HIV....I heard of the testing once ....” (P12: 19 years old).

“I do my work every day and physically feel well, I don’t need that test. I think people would like to take a health test if they do not feel well or get sick for a certain period of time” (P27, 32 years old).

“I know about the test but why would I take the [HIV] test if I am not sick? It is weird if I am not sick but taking the test, it would make people assume that I have done something wrong ....” (P25: 23 years old).

“I do not test for it [HIV] because I do not think I can get the infection. I am fine, I feel great. Besides, I do not easily get sick even though my work is physically demanding. I work for the entire day every day, drive passengers from one place to another ....” (P38: 31 years old).

Fear of a positive result of HIV testing was another reason supportive of the unwillingness of the clients of FSWs to access HIV testing services available for them. Ten interviewees commented that knowing HIV positive status could make them feel stressful, worried, and depressed; hence not taking HIV testing seemed to be a reasonable choice for them. Some of the assertions indicative of participants’ fear of knowing their HIV status included:

“I once thought of undergoing HIV testing but I am afraid of the result. It would be very stressful if I am positive. I have seen this on one of my neighbours who is HIV positive, he is stressful. I do not want to experience that” (P1: 25 years old)

“I am not afraid of the [HIV] test but worried about the result. I do not know what to do if I get infected. This makes me feel not ready yet to take the test” (P14: 27 years old).

“When the time comes then I will do it [take HIV testing]. I do not want to do it now because I do not want to feel depressed if the result is positive” (P30: 24 years old).

“I do not undergo HIV test because I am scared if I am positive. I think I need to really prepare myself if I want to take the test. It will be a difficult decision because the result can change my life personally and socially” (P2: 22 years old).

### Health care service provision barriers

#### Lack of health service trust in fear of disclosure of their HIV status by health professionals

Lack of trust in health professionals was discovered to also influence the decision of FSWs’ clients to undergo HIV testing as depicted in nine participants of varying ages. It was suspected that HIV status of people living with HIV/AIDS was made known to public by health professionals. As the consequence, the clients of FSWs did not want to access HIV testing service because they did not trust the health professionals in keeping the secret of the test result or their HIV status:

“I do not test because I do not trust their mouths [health professionals]. They would tell their friends and family members if they know someone is infected with HIV” (P8: 32 years old).

“I know they [health professionals] do not keep the secret of the health condition of patients. If I want to test for HIV, I would go to do it somewhere else but not here” (P22: 35 years old).

“Information about HIV positive people spreads so fast here, I am suspicious they [health professionals] are the ones who spread it. This is the reason I do not want to get tested” (P37: 24 years old).

“I do not accuse them [health professionals] but I guess they would tell their families or friends if they know that someone is HIV positive. .... I do not want to test [for HIV]” (P24: 23 years old).

### Limited availability of medications including antiretroviral (ARV)

Limited availability of ARV also emerged during the interviews. It was brought up by two participants, commenting that ARV was hardly available in the districts and HIV/AIDS patients did not get treatment. This was indicated to be another factor that influenced them not to undergo HIV testing:

“I have thought of testing for HIV but I have not yet done it because I heard that ARV is very limited here. It would be stressful if I get infected but there is no ARV to treat the infection. I know a few HIV/AIDS positive patients do not take ARV because it is not available” (P16: 29 years old).

“Knowing or not knowing my HIV status makes no different to me because even if I am HIV positive I cannot access the treatment regularly because ARV is very limited. I know it is not available in the community health centres” (P31: 26 years old).

Fourteen study participants who had been exposed to information on HIV/AIDS confirmed that treatment to HIV/AIDS or ARV was made available only in one hospital. These participants appeared to know that patients diagnosed with HIV infection in community health centres should receive further treatment at that hospital. This further step needed for accessing ARV seemed to be an additional barrier for voluntarily HIV testing, as could mean that if diagnosed, it would be a process before receiving the required treatment. Testifying assertions included:

“ARV is only available in general hospital of Belu, patients diagnosed with HIV in a community health centre should go the hospital in the city to get the medicine or treatment, this discourages me to take the test ....” (P9: 26 years old).

“Even though someone is tested positive in a community health centre, he or she has to access HIV/AIDS treatment or ARV at the hospital [general hospital of Belu] because treatment for HIV/AIDS is not available at community health centres. That means if I take the test and get diagnosed with the infection, then I need to go the hospital every time I feel sick and people will notice me” (P19: 21 years old).

“I know about HIV testing but one of the reasons that discourages me to do the [HIV] test is the limited availability of ARV. I once read in a newspaper that ARV was not available at the general hospital of Belu for more than six months” (P42: 27 years old).

“It is difficult to access ARV. HIV patients should go the hospital that provides ARV .... It [the hospital] is quite far from here” (P10: 30 years old).

It was interesting that the cost required for medication and transportation to the health services points seemed to not be barriers to accessing HIV testing among the study participants. Twenty seven participants across age and work group categories commented that they could afford the medical and transportation costs as indicated in the assertions below:

“.... I can pay for health service or buy medicine, I guess it is not a problem. .... I just do not know about it [HIV testing], haven’t heard of it before” (P6: 20 years old).

“Is there a test for the disease [HIV infection]? Maybe I can try sometime .... I can drive to the community health centre or hospital, I have a motorbike .... the community health centre is not far from here” (P17: 30 years old).

“.... I don’t think it would be difficult for me to access the service [HIV testing], every day I pass through the community health centre. I don’t worry about the buying medicine or the transport .... As I told you before, I haven’t heard of anything about the [HIV] test or the disease [HIV infection] ....” (P21: 22 years old).

“.... I guess maybe it is because I never think of getting tested for HIV .... No, I don’t think the costs to travel to community health centre or to purchase medicine are a big deal for me....I do not do it because I do not know at all ....” (P26: 33 years old).

### Social barriers

#### Fear of stigma and discrimination

HIV/AIDS-related stigma and discrimination are still prevalent in the districts where the current study was conducted and in Indonesia as a whole. Fear of being stigmatised and discriminated as the result of both accessing HIV testing service and being an HIV positive person seemed to be another precluding reason for the clients’ HIV testing. This not only kept them away from accessing HIV testing service to know their HIV status but also other necessary HIV-related services, hence making them even more vulnerable to HIV infection and its impacts:

“If I go and access the service [HIV testing] or take the [HIV] test then people who see me or know about it would think that I am [HIV] positive and start gossiping about my HIV status. People are very sensitive about this issue [HIV], I have heard of people talking about the HIV status of others who did the HIV counselling and testing” (P18: 21 years old).

“I do not want to be stigmatised. Everybody seems afraid of HIV, they would look at me with one eye [cynical] if they know I do the [HIV] test. This has often happened to many HIV positive people and I have seen it with my own eyes. I do not want the same thing happens to me” (P23: 26 years old).

“I do not want to do it [HIV testing] because people will start staying away from me if they know I do it [take HIV testing] or if I am [HIV] positive. I think people do not want to get close to or make any physical contact with HIV positive ones because they do not want to get infected with HIV ....” (P29: 24 years old).

“Stigma against people living with HIV/AIDS is still prevalent here, that is why I do not want to know my HIV status. If I do the test and the result is positive, then sooner or later people will know it. They will start avoiding me, gossiping about me and this will worsen my condition ....” (P36: 28 years old).

### Lack of social supports from friends and family

The lack of social support played a role in influencing health seeking behaviour of the clients of FSWs. It appears that all the study participants did not receive support including information and emotional supports from friends or family members or health professionals to undergo HIV testing or access HIV/AIDS-related services. This might be due to the topics of HIV/AIDS and HIV testing were absent from their daily conversation with friends and family members, and they did not consult health professionals either:

“I do not feel any support from friends or family in relation to HIV testing. We never talk about this topic” (P5: 28 years old).

“My parents, brothers and sisters do not know about my sexual behaviour, so they never say anything about HIV/AIDS or HIV testing. I think they do not understand about HIV/AIDS or HIV testing” (P13: 33 years old).

“None of health professionals tells me or encourages me to undergo HIV testing. I would do it if a doctor or nurse helps me and if I am convinced that it is safe and confidential. But to be honest, I never seek for help from health professionals” (P34: 25 years old).

## Discussion

This study aimed to identify barriers to accessing HIV testing services among clients of FSWs in two Indonesian districts. The engagement of clients of FSWs in sex with multiple sexual partners without using condoms reported in previous findings in Indonesia and elsewhere may put them at high risk for HIV infection [7–10, 35]. The findings of the current study reveal that despite having multiple sex partners and engaging in unsafe sex, all the participants had never had an HIV test at the time of this study. As such, they did not know their HIV status for further appropriate action. Access to HIV testing services is an important finding which is well acknowledged to be central to HIV diagnosis and an important part of HIV care continuum [20, 23]. Although the HIV testing services were **available and affordable** (free of charge) in the study settings, they were not necessarily easily accessible to clients of FSWs in the study districts. The lack of knowledge about the availability of these services and low self-perceived risk for HIV infection which are in line with previous studies’ findings [3, 12] were the main barriers for the majority of the clients of FSWs to accessing the services. As purported by the health accessibility framework [23], some of the participants abilities as well; for example, the ability to seek (e.g. due to cultural and personal values) and the ability to reach (e.g. due to distrust of the professionals) depicted in the responses of some participants, seemed to influence the decision to access HIV test service by the study participants. Some of these aspects, including limited availability of healthcare facilities, poor affordability of medicine and travel to health care facilities, and lack of knowledge of HIV problem, have also been reported in previous studies as hindrances to accessing HIV/AIDS-related health services [36–38].

As previously noted ‘the idea of knowing’ (a positive diagnosis) of the HIV status by individuals creates fear, and is a known barrier to accessing services [19, 21]. After nearly 40 years after the initial emergence of HIV knowledge, the current study indicates that the fear of an HIV diagnosis was still a hindrance to accessing HIV testing services by the participants. Further, Lahuerta and colleagues [21] and Darling and colleagues [19] have cited that HIV stigma and discrimination occur as the consequences of an HIV positive diagnosis in some populations. Stigma and discrimination may relate to poor **approachability** of service providers and **poor appropriateness** of health care service provision [23]. In this study, these were depicted in the participants’ distrust of the health professionals – with the perceptions that health professionals were capable of breaching confidentiality of the HIV test results. Findings from studies elsewhere have shown that the lack of anonymity and confidentiality of HIV test results are important decision drivers to HIV testing [36, 39–42]. It is important to note that although stigma and discrimination were noted to be barriers to accessing HIV/AIDS-related health services in other population groups [43–45], in the current study, stigma and discrimination expressed by the study’s participants were perceived rather than actual, as none of them had accessed HIV testing services or diagnosed with HIV infection. In supporting the access framework and studies elsewhere [3, 23, 24], poor **availability** of ARV for HIV treatment was an additional barrier to undertaking the HIV testing for a few study participants whose perceptions were that diagnosis would not necessarily lead to HIV treatment required in the HIV care continuum.

Other socio-cultural issues including the absence of HIV/AIDS and dialogues in daily conversations, and the lack of support from families, friends, health professionals and significant others, since the emergence of HIV scourge ~ 40 years ago [46], leave a lot to be desired. It is well established that social support from peers, sex partners and families, including caring attitudes, accompanying to healthcare facilities for testing, and helping with transport to health facilities increases motivation for HIV testing and reduces HIV testing-related anxiety [37, 47, 48]. It is plausible to indicate that the motivation to improved testing availed via social support might be a result of the lessening of fear of being avoided or rejected by significant others, known to affect people living with HIV, and as stated elsewhere, a barrier to HIV testing [47, 49, 50]. Trust in significant others and facilitators of HIV testing such as the provision of incentives, implementation of HIV testing at community or household level and at private testing facilities and provider-initiated HIV testing which have been reported elsewhere [38, 49, 50], were not mentioned by participants. Given that there was previous reporting of the existences of active FSWs in the study settings [24, 25], the current study presents important results indicating the urgency to promote HIV testing for both the clients of FSWs and the FSWs in the study districts as a strategy to prevent and/or reduce HIV infection in FSWs and their clients as well as for the general community in the settings and across Indonesia.

### Limitations and strengths of the study

The findings of the current study should be interpreted with caution due to several limitations. Firstly, the study participants were recruited from only two settings that have similar characteristics including: socio-cultural and religious norms and values, HIV/AIDS-related health services, procedures to access health services, and the dissemination of knowledge and information of HIV and the existence of HIV related services. As such, the findings of the current study reflect the situation of the study participants in the study settings, which might be different to similar clients in other settings with different characteristics. The use of snowball sampling technique could have also been a limitation as it might have resulted in underrepresentation of participants outside the social networks of the current study participants or from other parts or outside of the districts, leading to an incomplete overview of the barriers to accessing HIV testing among the clients. It is therefore reasonable to be cautious and to assume that the current study findings are less likely to be transferred to clients from different sites with different characteristics. However, to our knowledge these are the first qualitative study findings on this topic, hence could be useful to inform HIV strategies and interventions for this group in the study settings or other similar settings in Indonesia.

## Conclusions

This study reports several barriers to accessing HIV testing services among clients in the study settings. Three levelled barriers including at: (i) personal, (ii) health care service provision, and (ii) societal levels, were found. The lack of knowledge about the availability of HIV testing services was a major barrier for HIV testing among study participants. However, the clients who were aware of the HIV infection and the service did not undergo HIV testing either. Limited availability of HIV treatment including ARV and perceived stigma and discrimination after being known to be HIV positive, were significant underlying reasons for participants to not access the HIV testing services. To improve the accessibility to HIV testing and overall health outcomes by clients, a multilevelled approach is needed to address all the barriers. These may include: the dissemination of information on HIV/AIDS and improved availability of HIV/AIDS to improve the personal levelled barriers. The system wide barriers will need improved practices and health policies, particularly in providing patient-friendly and accessible services. The societal levelled barriers will need a more broad societal approach, including raising awareness in the community and enhanced discussions about HIV/AIDS issues in order to normalise HIV in the Indonesian society. In addition, further studies with a larger number of heterogeneous participants from different sites especially urban areas are recommended as the results of such studies can be transferable to larger communities in Indonesia and other similar settings.

## Abbreviations

AIDS: Acquired immune deficiency syndrome
ARV: Antiretroviral
FSWs: Female sex workers
HIV: Human immunodeficiency virus
